# Periodontitis and diabetes: a bidirectional link

**DOI:** 10.1007/s00592-026-02642-3

**Published:** 2026-02-02

**Authors:** Thanh T. Nguyen, Miguel Bandeira, Catherine Giannopoulou, Alkisti Zekeridou, Dongryeol Ryu, Karim Gariani

**Affiliations:** 1 Department of Biomedical Science and Engineering, Gwangju Institute of Science and Technology, Gwangju, Republic of Korea; 2 Center of Endocrinology, Metabolism, Genetic/Genomics and Molecular Therapy, Vietnam National Children’s Hospital, Hanoi, Vietnam; 3 Division of Regenerative Dental Medicine and Periodontology, University Clinics of Dental Medicine, University of Geneva, Geneva, Switzerland; 4 Division of Endocrinology, Diabetes and Metabolism, Department of Medical Specialties, Geneva University Hospitals, Geneva, 1205 Switzerland; 5 Faculty Diabetes Center, University of Geneva Medical Center, University of Geneva, Geneva, Switzerland

**Keywords:** Diabetes, Periodontitis, Oral health, Oral inflammation, Cardiovascular

## Abstract

Periodontitis is a chronic inflammatory disease affecting the tooth-supporting structures, and its closely linked to diabetes mellitus through a well-established bidirectional relationship. Diabetes exacerbates periodontal destruction via systemic inflammation, oxidative stress, and immune dysfunction, while periodontitis can impair glycemic control by increasing systemic inflammatory burden. The pathogenesis of periodontitis remains only partially understood, involving microbial dysbiosis, host immune responses, and metabolic disturbances. The 2018 classification system defines stages and grades based on disease severity and progression risk. Epidemiological data reveal a high global prevalence, particularly among individuals with type 2 diabetes. Studies have shown that periodontal therapy contributes to improved glycemic control and may reduce cardiovascular risk. Despite its clinical significance, periodontitis remains underdiagnosed in the context of diabetic care. Effective management requires integrated medical and dental collaboration, targeting both glycemic regulation and periodontal health. This dual approach offers mutual benefits for reducing complications and improving long-term outcomes in diabetic patients. In this review, we present the current knowledge on the relationship between diabetes and periodontitis, focusing on epidemiology, pathogenesis, and management.

## Introduction

**Fig. 1:**
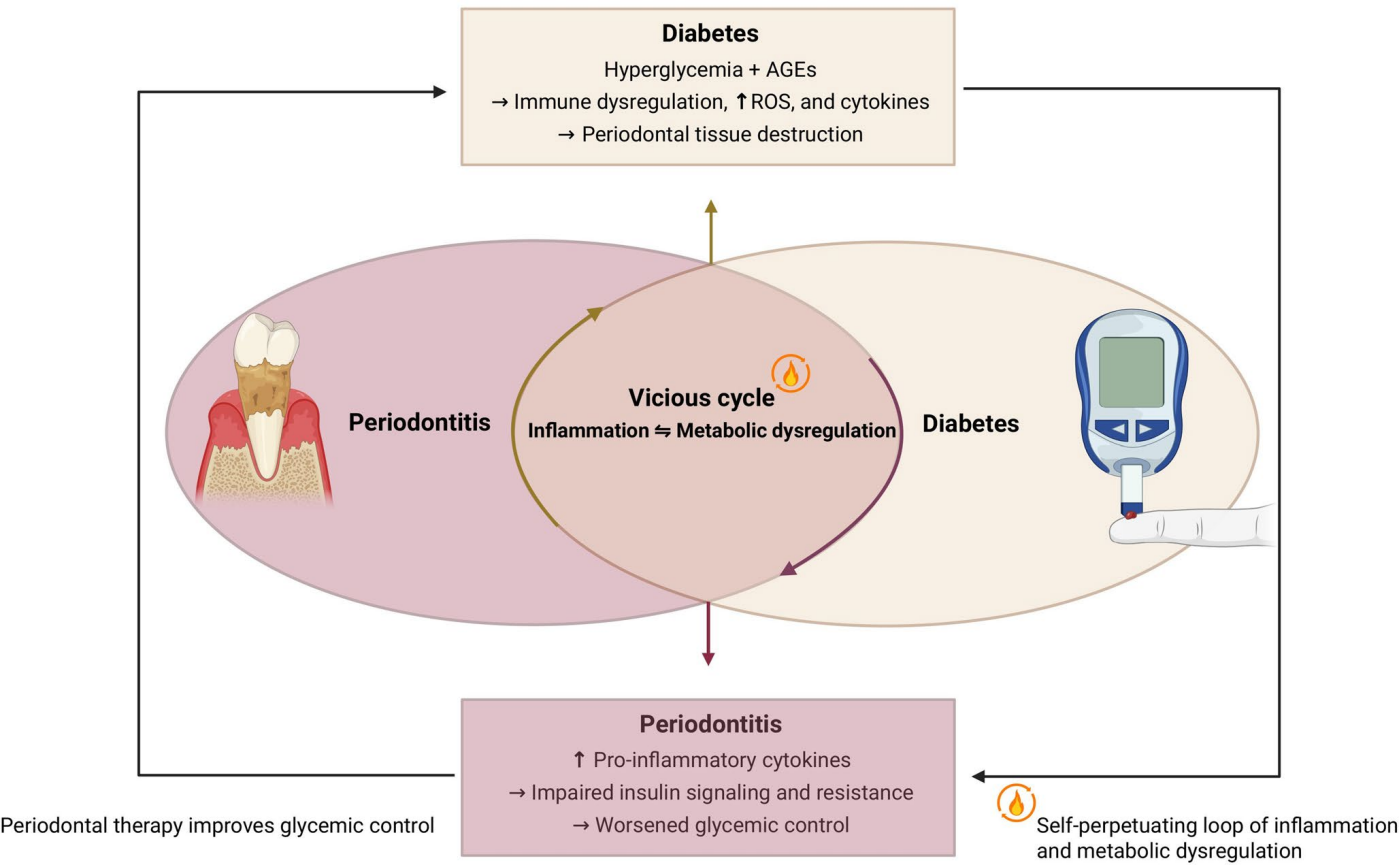
The Bidirectional Relationship Between Diabetes and Periodontitis. Diabetes and periodontitis exhibit a bidirectional relationship. Poorly controlled diabetes leads to hyperglycemia, the formation of advanced glycation end-products (AGEs), and activation of inflammatory pathways, which contribute to the destruction of periodontal tissues. Conversely, periodontitis induces local and systemic inflammation that disrupts glucose homeostasis, thereby exacerbating diabetic conditions

Periodontitis is a highly prevalent chronic inflammatory disease affecting the supporting structures of the teeth and is increasingly recognized for its systemic implications. Among the numerous systemic associations, the link between periodontitis and diabetes mellitus stands out due to its strong bidirectional nature [1]. Chronic hyperglycemia contributes to the dysregulation of immune responses and exacerbates periodontal tissue destruction, while periodontitis itself may impair glycemic control by sustaining systemic inflammation [2]. Despite over three decades of evidence, periodontitis remains an underappreciated complication of diabetes, often omitted from routine care protocols. The pathogenesis of periodontitis is multifactorial and only partially elucidated, involving microbial dysbiosis, host inflammatory pathways, and genetic-environmental interactions [3]. Given its significant clinical and public health burden, particularly in diabetic populations, a deeper understanding of this interaction is critical. Integrated care strategies targeting both periodontal health and glycemic control may offer reciprocal benefits, highlighting the need for interdisciplinary collaboration in managing these interconnected conditions (Fig. 1).

## Definition and classification

**Fig. 2:**
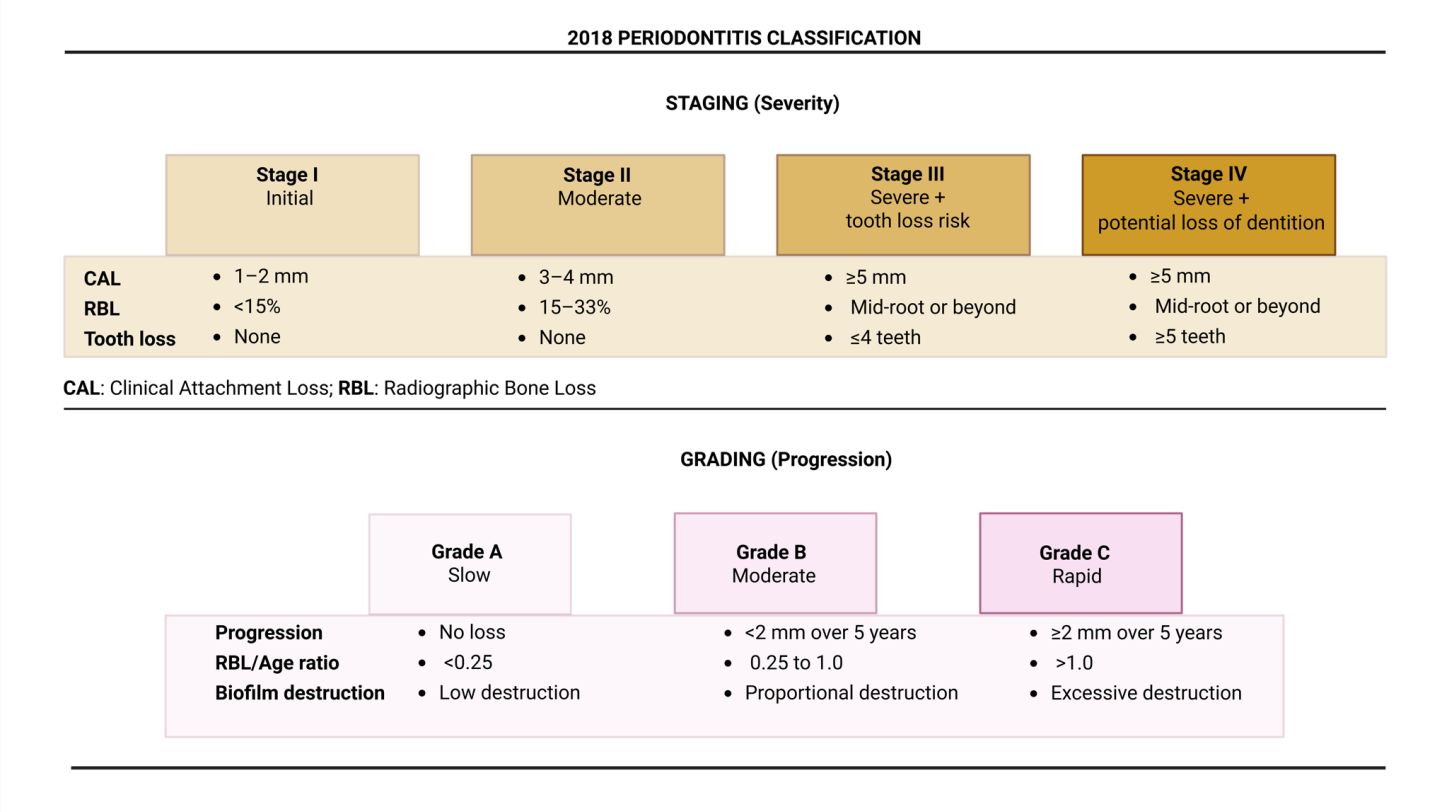
Diagnostic Framework for Periodontitis Based on the 2018 Staging and Grading Criteria. Overview of the 2018 periodontitis classification, illustrating staging based on disease severity (clinical attachment loss, radiographic bone loss, and tooth loss) and grading according to progression rates. Stages I–IV reflect increasing tissue destruction and risk of tooth loss, while Grades A–C indicate slow, moderate, or rapid progression. This framework supports comprehensive diagnosis and personalized treatment planning

Periodontitis is defined as a chronic inflammatory disease of periodontal tissue leading to the progressive destruction of alveolar bone and periodontal ligament that may result in tooth loss. Periodontitis has now been considered for more than 30 years as a full-fledged complication of diabetes and is also called the “6th complication of diabetes” after retinopathy, nephropathy, neuropathy, cardiovascular diseases and peripheral arterial disease. Periodontitis is a public health problem whose high prevalence contributes to the global burden of chronic non-communicable diseases. The classification of periodontitis is currently based on the consensus report of 2018 [4] Compared to the previous version of 1999 [5], , the 2018 version has made several modifications based on updated knowledge of this disease. A case of periodontitis is now defined as the presence of interdental clinical attachment loss (CAL) at two or more non-adjacent teeth, or buccal/oral CAL of ≥ 3 mm with pocketing of ≥ 3 mm detectable at two or more teeth, provided that the CAL is not attributable to non-periodontitis-related causes, such as dental caries extending into the cervical area of the tooth or gingival recession of traumatic origin. In the 2018 consensus, periodontitis was also reclassified into three types based on pathophysiology—necrotizing periodontitis, periodontitis, and periodontitis as a manifestation of systemic disease—and a staging and grading classification system was introduced. The staging system is divided into four levels: **Stage I**, characterized by early attachment loss; **Stage II**, representing established periodontitis; **Stage III**, marked by significant damage to the periodontal attachment; and **Stage IV**, involving severe loss of periodontal support leading to tooth loss and impaired masticatory function. The grading system classifies the risk of periodontitis progression, the expected clinical response to therapy and potential interactions with general health. It emphasizes factors influencing periodontal disease that are not directly related to the current severity of tissue damage. The grading classification is divided into three levels: **Grade A**, where disease progression is minimal or arrested; **Grade B**, indicating a moderate rate of progression; and **Grade C**, reflecting a high risk of rapid disease progression (Fig. 2) [6]. Validated screening tools play a crucial role in the early identification of periodontitis, enabling timely intervention and reducing the risk of advanced tissue destruction. As the 2018 classification provides a refined diagnostic framework based on staging and grading, reliable screening instruments are essential for detecting individuals who may require full periodontal assessment. Over recent years, several case definitions have been proposed, with the 2012 CDC/AAP criteria becoming the most widely used in epidemiological research and regarded by many as the gold-standard reference [7]. Tools derived from this system, such as the CDC–AAP Questionnaire, help identify at-risk individuals based on demographic, behavioral, and symptom-related indicators [8]. The IDF–EFP Oral Health Checklist further facilitates early detection in medical settings, especially in diabetes care, by linking periodontal risk with systemic health and guiding referral [9, 10]. In clinical practice, the PSR Index, endorsed by the ADA and AAP and adapted from the WHO-validated CPITN, provides a quick, reliable, and reproducible chairside method to determine whether comprehensive periodontal evaluation is warranted [11, 12]. Together, these tools strengthen early detection pathways and support a more integrated approach to periodontal care.

## Epidemiology

Periodontal disease is a condition with a very high prevalence for several decades. According to the Global Burden of Disease (GBD) study, periodontitis was identified as one of the most common health conditions worldwide from 1990 to 2010 [13, 14], and a recent update through 2019 reaffirmed that its prevalence remains significantly high, which is a notable public health concern [15]. A systematic review/meta-analysis including a total of 55 studies carried out in 17 different countries reported an overall prevalence estimated at 62% and a prevalence of around 24% in particular for severe periodontitis [16]. The prevalence of periodontitis in the general population varies between included studies, probably linked to the use of different definitions and diagnostic criteria [17].

The prevalence of periodontal disease among specifically diabetic individuals has been evaluated in several studies. The prevalence appears to differ between individuals with type 1 or type 2 diabetes. In the case of type 1 diabetes, a recent meta-analysis reported a prevalence of approximately 18.5%, with an odds ratio of 2.5 compared to the general population [18].

In the case of type 2 diabetes, the prevalence of periodontal disease seems higher, with estimated values around 70 to 80%, with even some studies reporting prevalences greater than 90% [19–21]. A meta-analysis including a total of 53 studies showed that type 2 diabetes increases the risk of developing periodontitis by 34% and is linked to greater disease severity. Indeed, in the case of periodontal disease, the presence of type 2 diabetes is associated with two additional missing teeth, greater attachment loss, and deeper periodontal pockets [22].

A certain number of risk factors have been identified for periodontitis including age, low socio-economic status, diabetes, obesity, unhealthy diet, smoking, alcohol consumption, substance use disorders, mental health disorders, rheumatoid arthritis and some medications such as calcium channel blockers, phenytoin or ciclosporin [23, 24].

## Pathogenesis

Periodontitis is caused by a combination of periodontal microbiome dysbiosis and the host immune response. The relationship between periodontal disease progression and the oral microbiota is complex and remains only partially elucidated and is influenced by both genetic and environmental factors. Several key structures contribute to the function and attachment of teeth, including the alveolar bone, alveolar mucosa, gingiva, cementum, and periodontal ligament.

### Diabetes and oral dysbiosis

The periodontal microbiome represents a significant portion of the entire microbiome and includes hundreds of distinct species. The balance between the commensal microbiota and the host plays a fundamental role in oral health and modifications in terms of the composition of the oral microbiota will lead to dysbiosis favouring the development of periodontitis. The formation and development of oral biofilm appear to be a major factorsin the pathogenesis of periodontitis and are induced by proliferation and coaggregation of microorganisms. The accumulation and expansion of multiple biofilm plaques on the tooth surfaces and within periodontal pockets promote the development of inflammation, which can ultimately lead to tooth loss [25]. Among the potential pathogenic bacteria identified in periodontitis, *Porphyromonas gingivalis* is at the top of the list [26].

It has been shown that the oral microbiome of diabetic individuals with or without periodontal disease is different from non-diabetic people, notably with a significant reduction in biological and phylogenetic diversity [27, 28]. In addition, in the case of clinical periodontics, the oral microbiome differs in the case of associated or unassociated diabetes with, in the case of diabetes, a greater abundance of various species such as *Corynebacterium*, *Lactobacillus* or *Saccharibacteria* and a reduction of others such as *Prevotella* or *Filifactor* [29]. In the presence of diabetes, the composition of the oral microbiome also seems to be influenced by the degree of glycemic control, particularly in the case of higher HbA1c, a reduction in diversity and a greater proportion of species using carbohydrates such as *Veillonellaceae* and *Prevotellaceae* [30]. Despite advances such as next-generation sequencing (NGS), the structural complexity of the biofilm matrix and the diversity of oral microbiota species make it difficult to precisely determine the role of each microbial component in periodontitis, particularly in relation to the presence or absence of diabetes. It nevertheless remains established that the colonization of the oral cavity by a resident microbiota is beneficial in particular for preventing the emergence of pathogenic species but the presence of an excessively significant inflammatory response contributes to the destruction of bone tissue and also to the development of the oral dysbiosis [31].

### Oral microbiota and inflammation

The development of oral dysbiosis leads to the development of inflammation which is mainly mediated by the interaction between complements and pattern recognition receptors (PRRs) [32]. This inflammation gradually leads to the destruction of periodontal tissues and bone loss, which in turn releases proteins and other nutrients from the damaged structures, providing a nutrient source for the microorganisms responsible for dysbiosis [33]. In the specific case of *P. gingivalis*, the signaling pathways have been identified quite precisely. Initially, interaction between *P. gingivalis* and Toll-like receptor (TLR2) and the action of the bacteria lead to the production of the complement components C5a and C5b [26]. The C5a ligand then binds to its C5ar receptor initiating a cascade that promotes the differentiation of myeloid cells, ultimately amplifying inflammation and exacerbating the progression of periodontitis [34].

Although the contribution of diabetes to the pathogenesis of periodontitis is well established, its impact on the development of oral dysbiosis remains a matter of debate. Technological advances, notably NGS, have made it possible to better study the composition and modifications of the oral microbiome in patients with or without diabetes.

**Fig. 3:**
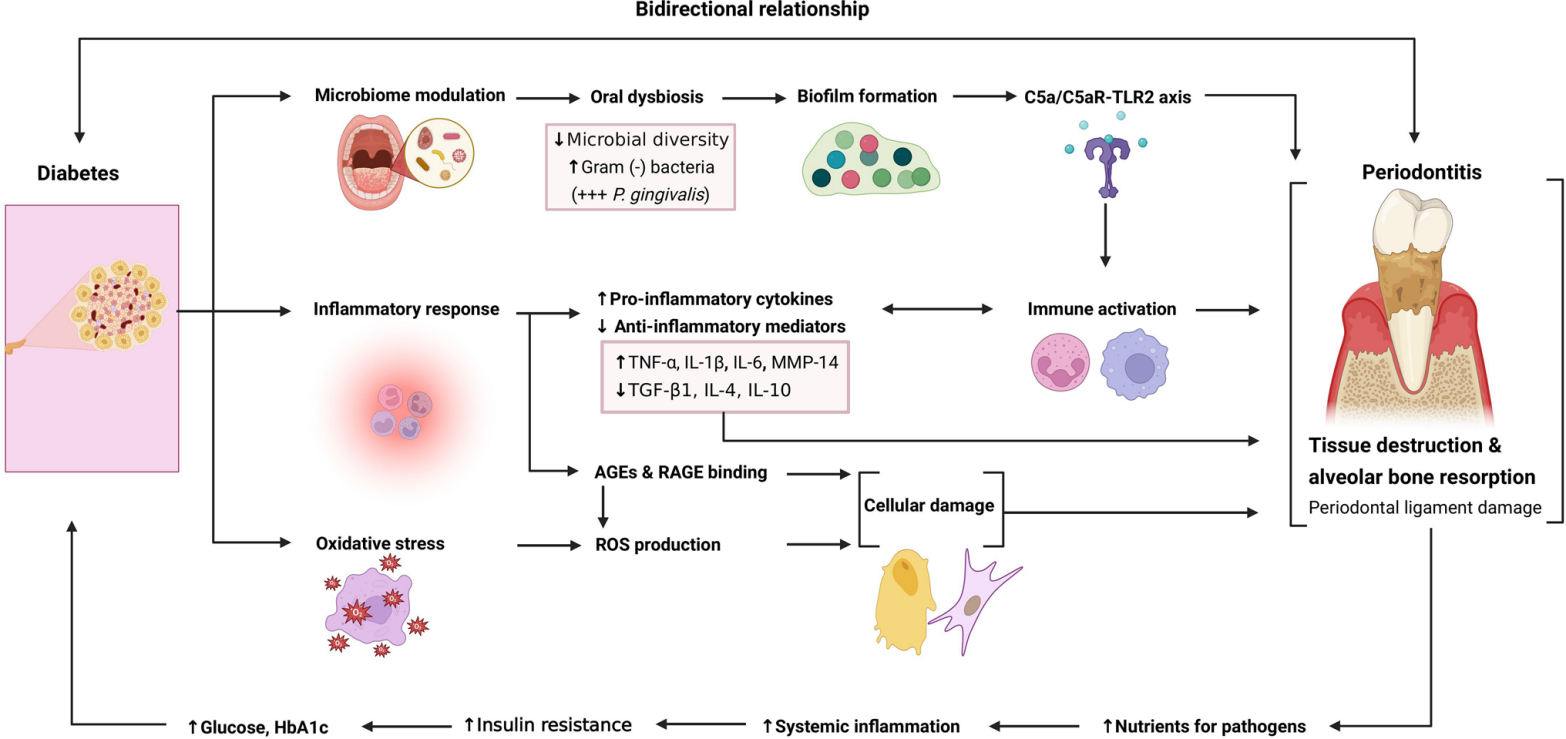
Pathophysiological Mechanisms Linking Diabetes and Periodontitis. The interplay between periodontitis and diabetes involves complex mechanisms. Poorly controlled diabetes disrupts the oral microbiome, activates complement pathways, promotes biofilm formation, and elevates oxidative stress, triggering an inflammatory response with cytokine release. Periodontitis leads to destruction of periodontal tissues, ligament degradation, and alveolar bone resorption, perpetuating local and systemic inflammation

The exaggerated inflammatory response involved in the pathogenesis of periodontitis is particularly marked in cases of diabetes. We observe an increase in the expression levels of various cytokines such as Tumor necrosis factor-alpha (TNF-α), Interleukin-1 beta (IL-1β) or Interleukin 6 (IL-6), with a reduction in the levels of anti-inflammatory elements such as TGF-B or IL4 or inflammatory regulatory T cells (Fig. 3). The increase in the expression levels of these cytokines results in the arrival of inflammatory cells in the periodontal tissue and an increase in the permeability of blood vessels, promoting periodontitis and bone destruction [35]. Other mediators, such as MMP-14 and substance P, contributing to a pro-inflammatory state, have been found at higher levels under hyperglycemic conditions in gingival crevicular fluid or periodontal tissues, along with a decrease in anti-inflammatory mediators such as lipoxins and protectins [36, 37].

### Hyperglycemia and oxidative stress

The hyperglycemic state causes the formation of advanced glycation end products (AGEs) as well as that of reactive oxygen species (ROS), which maintain the local inflammatory state and the progression of periodontal disease. Among the currently identified factors linking oxidative stress and periodontitis in the context of diabetes, available data remain limited but suggest an association with decreased expression of the transcription factor Nrf2 in severe periodontitis, along with increased levels of biomarkers such as 4-hydroxy-2-nonenal and 8-hydroxydeoxyguanosine, indicating a local exacerbation of oxidative stress; furthermore, diabetes itself is associated with elevated levels of malondialdehyde (MDA) and reduced superoxide dismutase activity, reflecting increased systemic oxidative damage and diminished antioxidant capacity, thereby supporting a potential central role for oxidative stress in the bidirectional relationship between diabetes and periodontitis, characterized by mutual activation and worsening [38]. In this context, mitochondrial dysfunction has emerged as a critical contributor to oxidative stress and inflammatory amplification in diabetic periodontitis [39]. Hyperglycemia-induced mitochondrial alterations promote excessive mitochondrial ROS production, impaired energy metabolism, and dysregulation of antioxidant defense systems, further aggravating periodontal tissue damage. Moreover, mitochondrial-derived ROS and mitochondrial DNA damage may activate inflammatory signaling pathways, reinforcing the chronic inflammatory microenvironment [40]. These mechanisms not only exacerbate periodontal destruction but may also impair tissue repair and regenerative capacity. Therefore, targeting mitochondrial dysfunction represents a promising avenue for improving therapeutic strategies in diabetic periodontitis [41].

Many gaps remain to be filled in the understanding of the pathogenesis of periodontitis in connection with diabetes and the advent of technological approaches such as metabolomics, proteomics or virome with a solid bioinformatics methodology could make it possible to advance further and potentially to identify specific therapeutic targets [25].

## Periodontitis and cardiovascular disease

Diabetes is associated with a two- to fourfold increased risk of cardiovascular diseases such as coronary artery disease (CAD), ischemic heart disease, heart failure (HF), stroke, and peripheral artery disease (PAD), which together account for over 50% of deaths among individuals with diabetes, making them the leading cause of mortality in this population [42].

The elevated cardiovascular risk observed in individuals with diabetes results from a multifactorial interaction that involves both classical risk factors, such as dyslipidemia and hypertension, and non-traditional contributors linked to the development of atherosclerosis. Among these, endothelial dysfunction and chronic inflammation play particularly prominent roles. Periodontitis, being a chronic inflammatory disease, contributes not only to local tissue inflammation but also to systemic inflammatory responses. This broader inflammatory burden has been suggested to enhance the risk of cardiovascular complications, particularly in diabetic patients, by promoting atherogenic processes [43]. Several works indicates a bidirectional and synergistic relationship among diabetes mellitus, periodontal disease, and cardiovascular disease, driven by chronic inflammation, immune dysregulation, oxidative stress, and microbial dysbiosis. Shared biomarkers, including IL-6, TNF-α, and CRP, and overlapping genetic and epigenetic signatures, suggest a common inflammatory axis linking these conditions [44]. Recent studies emphasize the importance of integrated management strategies, including periodontal therapy, cardiometabolic drugs, probiotics, and AI-based risk models, to mitigate systemic inflammation and improve clinical outcomes [45].

At the physiological level, bacterial colonization of dental plaque can lead to transient episodes of bacteremia. This allows bacterial components, including lipopolysaccharides, to reach the vascular endothelium. The detection of *P gingivalis* in atherosclerotic lesions provides strong evidence of this mechanism. It supports the hypothesis that periodontitis, especially when coexisting with diabetes, may facilitate the initiation and progression of atherosclerosis and related cardiovascular conditions [43]. Clinical and mechanistic evidence consistently supports that patients with poorly controlled diabetes exhibit more severe periodontal disease, while those with periodontitis experience impaired glycemic control [46]. Hyperglycemia-induced production of advanced glycation end products (AGEs) stimulates immune cells to release pro-inflammatory cytokines, such as IL-6, TNF-α, and IL-1β, which further damage periodontal tissues and promote insulin resistance. Moreover, periodontal pathogens like Porphyromonas gingivalis can translocate into systemic circulation, exacerbating endothelial dysfunction and contributing to atherogenesis. Interventional trials demonstrate that nonsurgical periodontal therapy improves glycemic control, reduces systemic cytokines, and may lower cardiovascular risk, underscoring the clinical relevance of managing these interconnected conditions [45, 47].

The local periodontal inflammation tends to propagate into the systemic circulation, establishing a persistent low-grade inflammatory state. This systemic inflammation contributes to endothelial dysfunction and activates immune and vascular cells. Increased activity of neutrophils and macrophages leads to elevated production of matrix metalloproteinases, while endothelial cells express higher levels of adhesion molecules such as VCAM-1 and ICAM-1, both of which are closely associated with atheroma formation [48]. In addition, the chronic inflammatory milieu stimulates the production of pro-thrombotic factors, including fibrinogen and plasminogen activator inhibitors, thereby increasing the likelihood of arterial thrombosis formation [49].

Several epidemiological studies have investigated the potential association between periodontitis in individuals with diabetes and mortality, particularly from cardiovascular causes. Notably, the Pittsburgh Epidemiology of Diabetes Complications study, which included over 300 individuals with type 1 diabetes, identified periodontitis as a significant predictor of coronary artery disease and major cardiovascular events, especially among current smokers [50].

A large-scale study involving approximately 4,300 individuals with diabetes, based on data from the NHANES database and conducted over more than 15 years of follow-up, found a significant association between periodontitis and both cardiovascular-related and all-cause mortality [51]. These findings suggest that the presence of periodontitis enhances long-term prediction of all-cause mortality and cardiovascular risk beyond traditional risk factors [51].

These epidemiological findings strengthen the biological connection between periodontitis, diabetes, and cardiovascular disease, highlighting the clinical and prognostic relevance of periodontitis in individuals with diabetes.

## Cost

It is challenging to accurately estimate the costs associated with dental care for patients with diabetes due to the highly variable healthcare systems, reimbursement structures, and access to dental services worldwide. The global annual cost of oral diseases for individuals with and without diabetes is estimated at $710 billion [52]. However, it remains challenging to determine the specific share attributable to individuals with diabetes.

Diabetes mellitus is associated with an elevated susceptibility to oral health conditions, potentially leading to increased healthcare expenditures. Empirical evidence indicates that individuals with diabetes demonstrate lower utilization rates of dental services, particularly regarding preventive care [53, 54].

Financial barriers appear to weigh more heavily on individuals with diabetes than on those without, particularly in the context of lower household incomes and limited access to dental insurance. These challenges are not merely theoretical; they manifest in measurable differences in healthcare utilization and costs. One study, for example, reported an increase of approximately US$19 per person per month in total healthcare expenditures among individuals with diabetes, illustrating how economic disadvantage compounds the burden of chronic disease [55]. By contrast, two other studies demonstrated cost reductions of US$75 and US$237 following periodontal treatment [56, 57]. However, the effect of periodontal care on diabetes-related costs remains uncertain, and there is limited empirical data from countries outside the USA, including the Netherlands.

Around 7 million adults in the United States, or one in six adults living with diabetes, postpone oral health care due to financial constraints [58]. This issue has significant practical and policy implications. On a personal level, failing to prioritize oral health, including routine professional cleanings and periodontal treatments, can have serious consequences for individuals with diabetes. These include the progression of periodontal disease, which can worsen insulin resistance and complicate diabetes management. In turn, poorly managed diabetes can accelerate the development of severe periodontal disease, potentially leading to tooth loss.

Several studies conducted across different countries and within diverse healthcare and reimbursement systems have consistently demonstrated that periodontal treatment in individuals with diabetes is associated with a reduction in diabetes-related healthcare costs. Non-surgical periodontal therapy, in particular, has been linked to improved glycemic control, a decrease in microvascular complications, and reduced productivity loss due to illness [59–61].

These findings support the notion that diabetic patients, especially those with poor oral health should be systematically offered periodontal care, not only to improve their overall health outcomes but also to achieve cost-effective and, in many cases, cost-saving benefits at both individual and healthcare system levels.

## Management

### Multifactorial approach

Prior to conducting any periodontal evaluation, a detailed medical history must be obtained to identify systemic conditions that may predispose the patient to periodontal disease. Comorbidities such as diabetes mellitus, rheumatoid arthritis, and tobacco use are well-established risk factors that can exacerbate periodontal breakdown and compromise treatment outcomes.

**Fig. 4:**
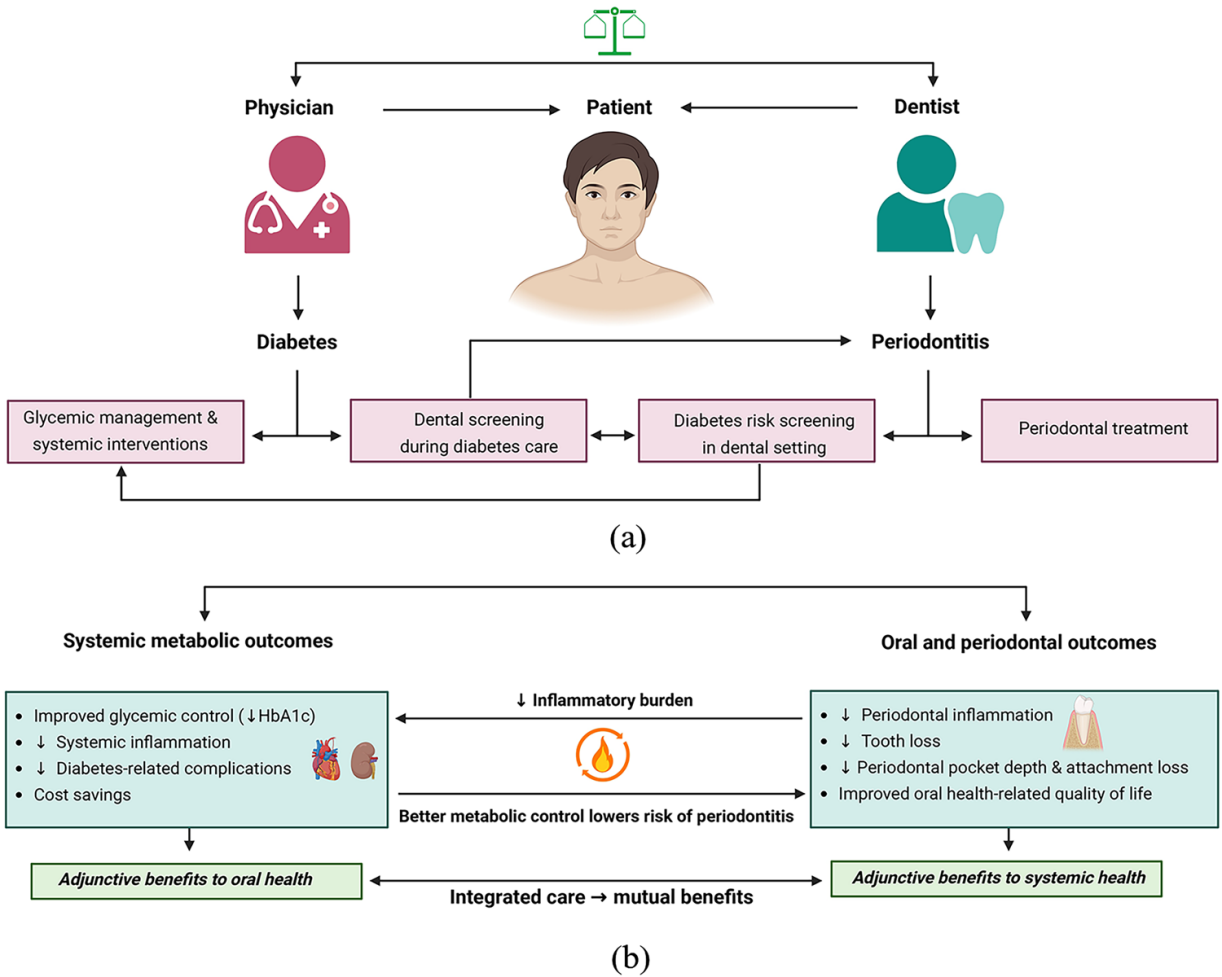
**(A)** Integrated Medical-Dental Care management pathway. Management of periodontal disease involves multiple aspects, including raising awareness among both patients and healthcare providers to promote regular dental screenings. When necessary, specific dental treatments combined with improved glycemic control are implemented to enhance both oral health and overall systemic health. **(B)** Pathophysiological and clinical benefits of integrated care. Improved metabolic control reduces systemic inflammation and diabetes-related complications, while periodontal care decreases periodontal inflammation and tooth loss. Reduced inflammatory burden underlies the mutual benefits of integrated medical–dental care

Effective management of periodontitis requires a multifaceted approach rooted in accurate diagnosis, causal elimination, and modification of risk factors (Fig. 4). A meticulous assessment of the patient’s periodontal status, including full-mouth probing, radiographic analysis, and evaluation of systemic health is the foundation upon which individualized treatment planning is based. Identification and control of modifiable risk factors, such as poor oral hygiene, smoking, uncontrolled diabetes, and other systemic inflammatory conditions, are integral to both disease control and long-term stability [62].

Initial therapy, typically non-surgical, focuses on patient education, behavioral modification, and mechanical debridement. This phase includes comprehensive oral hygiene instruction, reinforcement of effective plaque control techniques, and professional removal of subgingival and supragingival biofilm and calculus through scaling and root planing. Adjunctive antimicrobial therapies, whether systemic or local, may be considered in cases of young individuals with generalized severe disease or refractory disease [63]. Additionally, the use of host-modulation agents, such as sub-antimicrobial dose doxycycline, can be considered where indicated [64].

Once the initial inflammatory burden has been reduced, a re-evaluation phase is essential to determine tissue response and to identify residual sites with persistent pocketing, inflammation, or progressive attachment loss. In cases where non-surgical therapy proves insufficient, surgical intervention may be warranted. Contemporary regenerative surgical techniques, including the use of enamel matrix derivatives, bone grafts, and guided tissue regeneration, aim to restore lost periodontal structures. Alternatively, resective procedures, such as flap surgery or osseous recontouring, may be indicated to eliminate deep pockets and establish a more maintainable architecture [65].

Following active treatment, the patient transitions into a structured periodontal maintenance program. This phase is critical to ensuring long-term success and preventing disease recurrence. Maintenance visits typically occur at three- to four-month intervals and involve clinical monitoring, reinforcement of oral hygiene practices, and removal of new biofilm deposits. The frequency and nature of these appointments may be modified based on the patient’s individual risk profile and response to therapy [66].

Ultimately, the long-term retention of teeth in periodontally compromised individuals hinges on the sustained control of inflammation, ongoing patient compliance, and a strong partnership between the clinician and the patient. A well-implemented maintenance program, supported by patient education and risk factor management, is essential to preserve both periodontal health and quality of life [67]. As effective long-term maintenance relies on coordinated care and patient adherence, a clear delineation of the complementary responsibilities of general practitioners, diabetologists, and dental professionals is essential to support early detection and optimal management of periodontal disease. General practitioners serve as frontline providers, identifying systemic and behavioral risk factors, such as smoking, obesity, and poor glycemic control and facilitating referral for periodontal screening. Diabetologists play a key role in recognizing the bidirectional relationship between diabetes and periodontitis, integrating periodontal risk assessment into metabolic evaluations, and ensuring appropriate dental follow-up. Dental professionals then deliver definitive diagnosis, staging and grading, and individualized periodontal therapy while maintaining communication with medical providers. This coordinated, interdisciplinary model strengthens early detection, enhances personalized treatment pathways, and supports long-term periodontal stability [68, 69].

### Bidirectional relationship between periodontitis and diabetes treatment

A recent Cochrane review examined the impact of periodontal therapy on glycemic control in individuals with diabetes mellitus. The review included 35 randomized controlled trials, encompassing a total of 3,249 participants assigned to either periodontal treatment or a control group. All studies employed a parallel-group design and followed participants for periods ranging from 3 to 12 months. The findings revealed an absolute reduction in HbA1c ranging from 0.3% at six months to 0.5% at 12 months, suggesting a potentially meaningful clinical benefit [70].

Individuals with diabetes mellitus are two to three times more likely to develop periodontitis compared to non-diabetic individuals, a relationship demonstrated across numerous epidemiological studies [1, 71, 72]. These investigations have shown that diabetic patients exhibit a significantly higher prevalence and severity of periodontal disease than their non-diabetic counterparts [44, 73].

This increased susceptibility is primarily driven by the metabolic and immunological dysregulations associated with diabetes, including exaggerated inflammatory responses, impaired neutrophil function, altered microvascular integrity, and elevated oxidative stress, all of which contribute to the breakdown of periodontal tissues. Among the key factors influencing this risk, the degree of glycemic control plays a decisive role. Poor glycemic control has been linked not only to a greater likelihood of developing periodontitis but also to more severe and treatment-resistant forms of the disease. This mirrors patterns observed in other diabetes-related complications, such as retinopathy, nephropathy, and cardiovascular disease, where chronic hyperglycemia accelerates tissue damage.

Building upon this evidence, both non-surgical periodontal therapy (e.g., scaling and root planing) and surgical interventions aimed at controlling periodontal inflammation appear to exert beneficial effects on systemic metabolic parameters [74]. These treatments are thought to reduce systemic inflammatory mediators, such as TNF-α, IL-1β, and IL-6, which are known to interfere with insulin signaling pathways and contribute to insulin resistance [75, 76].

In the periodontal context, inadequate glycemic regulation exacerbates disease progression and fosters a systemic inflammatory milieu that may further impair insulin sensitivity, reinforcing a vicious cycle of metabolic and inflammatory dysregulation. These findings further strengthen the concept of a bidirectional relationship between diabetes and periodontitis, whereby each condition not only influences but also amplifies the severity and progression of the other. As such, effective management of periodontal inflammation in individuals with diabetes may not only improve oral health outcomes but also serve as a valuable adjunct in the broader strategy for preventing diabetes-related complications, including cardiovascular disease, nephropathy, and retinopathy [77].

Taken together, the available evidence supports the clinical relevance of integrating periodontal care into the comprehensive management of patients with diabetes mellitus. The consistent improvements observed in periodontal parameters, systemic inflammatory markers, and glycemic control following periodontal therapy highlight its potential role as an adjunctive strategy to conventional diabetes treatment [78, 79]. Although heterogeneity in study design, methodological quality, and the use of adjuvant therapies limits the strength of some conclusions, the overall trend indicates a beneficial bidirectional interaction. Future randomized controlled trials are needed to clarify the long-term metabolic effects of periodontal interventions and to identify patient subgroups most likely to benefit. Such efforts may contribute to more personalized and multidisciplinary approaches to the management of diabetes and its associated complications.

## Impact of periodontitis and chronic diseases beyond diabetes

Multidisciplinary working groups, including representatives from WONCA Europe and the European Federation of Periodontology (EFP), as well as the authors of a specially commissioned systematic review, have independently formulated evidence-based recommendations regarding the interrelationship between periodontal and systemic health. A central conclusion from these initiatives is the robust and independent association between periodontitis and a range of chronic non-communicable diseases (NCDs), including cardiovascular diseases, type 2 diabetes mellitus, chronic obstructive pulmonary disease (COPD), obstructive sleep apnea, and complications arising from COVID-19. Emerging evidence suggests that the successful management of periodontitis may contribute to measurable improvements in systemic health markers and disease outcomes, underscoring the bidirectional nature of these interactions [80].

Emerging evidence highlights a complex interplay between periodontitis, type 2 diabetes mellitus (T2DM), and COVID-19, with systemic inflammation emerging as a key mediator. Patients with poorly controlled T2DM are more susceptible to severe periodontal disease, which in turn can exacerbate hyperglycemia and systemic inflammatory burden. Periodontitis also facilitates the translocation of oral pathogens and enhances ACE-2 expression in oral and pulmonary tissues, potentially increasing SARS-CoV-2 infection severity. Concurrently, COVID-19 triggers a systemic cytokine storm, which can further aggravate periodontal inflammation and glycemic dysregulation [81]. This bidirectional relationship creates a vicious cycle where each condition amplifies the others, leading to worse clinical outcomes. Integrated management strategies targeting oral health, glycemic control, and infection risk are therefore essential to mitigate complications in patients affected by these three comorbidities [82].

Recent studies suggest that insulin resistance may also occur in the brain, driving neuroinflammation, impaired glucose metabolism, and the pathological changes characteristic of Alzheimer’s disease, an entity increasingly described as “type 3 diabetes.” Furthermore, Porphyromonas gingivalis, a key periodontal pathogen, has been implicated in exacerbating amyloid and tau pathology, with gingipain inhibitors showing promise in mitigating related neurotoxicity [83–85]. These findings broaden the systemic implications of periodontitis and reinforce the need to view oral health as an integral component of chronic disease prevention.

However, despite the high level of evidence supporting the relationship between periodontitis and diabetes, several surveys have shown that overall, people with diabetes have limited oral health knowledge, poor oral health attitudes and fewer dental visits [86–88]. On the other hand, most diabetes care providers do not address oral health care in consultations, with the main barriers being time constraints and limited knowledge in this area [89–91]. Given these findings, the call for integrated, collaborative care models is becoming increasingly urgent. Oral health care professionals (OHPs) and family physicians are encouraged to engage in coordinated management strategies that address both oral and systemic conditions. This includes the implementation of dual screening protocols for the early detection of periodontal disease within primary medical care settings, and conversely, the identification of systemic NCDs in dental clinics. Such interdisciplinary collaboration could enhance early diagnosis, facilitate timely intervention, and reduce the overall burden of chronic diseases at the population level [9].

However, the success of these initiatives is critically dependent on raising awareness among medical professionals, particularly general practitioners about the significance of periodontal diseases as both a public health concern and a modifiable contributor to systemic morbidity. There remains a pervasive underestimation of the clinical relevance of periodontal inflammation beyond the oral cavity. Educational efforts should therefore aim to integrate oral-systemic health concepts into medical curricula, continuing professional development programs, and public health campaigns.

Moreover, policy-makers and healthcare administrators must be engaged to support the inclusion of oral health indicators in general health records and to incentivize interprofessional collaboration. Only through sustained efforts in knowledge translation, professional education, and health system integration can the medical and dental professions work in concert to address the common inflammatory basis of many chronic diseases, improving patient outcomes across disciplines.

## Conclusion

Among the systemic conditions linked to periodontitis, diabetes mellitus occupies a particularly critical position due to its well-established bidirectional relationship with periodontal disease. Poor glycemic control has been shown to exacerbate periodontal inflammation, while effective periodontal therapy can contribute to improved metabolic control. This interplay highlights the necessity for integrated screening and management protocols involving both dental and medical professionals in the care of patients with diabetes.

Moreover, the pathogenesis of periodontitis is complex and only partially elucidated, involving a multifactorial interplay between microbial dysbiosis, host immune response, genetic predisposition, and environmental influences. In the context of diabetes, chronic hyperglycemia contributes to an exaggerated inflammatory response, further complicating periodontal tissue breakdown. Despite this well-documented association, periodontitis remains an often-overlooked complication of diabetes, underrecognized in routine medical care.

Comprehensive management therefore requires a dual approach: tight glycemic control to reduce systemic inflammation and immune dysfunction, alongside targeted periodontal treatment to reduce local bacterial load and inflammatory mediators. Addressing both conditions in tandem not only improves oral health outcomes but may also facilitate better long-term metabolic control, underscoring the importance of interdisciplinary collaboration in the care ofpatients with diabetes [10].
